# 

**Published:** 1871-12

**Authors:** 


### BOOK NOTICES.

ALL subscriptions for 1871 end with this number. Those who wish to renew, are requested to give notice, as it is proposed, as usual, to send the January number to those only who desire to take the JOURNAL OF HEALTH for another year. If each present subscriber would send another name with his own, it will be a kind of " Christmas gift " to the Editor, and will be an encouragement to him to enter upon another year's work.

" Flint's Practice of Medicine," 900 pp. 8vo, third edition, published in handsome style by Henry C. Lea, of Philadelphia, is now considered a standard work by educated physicians throughout the country. It is designed for the use of practitioners and students. As evidence of the intrinsic value of the work, the demand for it has been so general that, every year since its first issue, a new edition has been called for, and this third edition, thoroughly revised, brings up to the present time all reliable records of practice and progress, and is an indispensable volume to the young practitioner. The same publisher has an extensive assortment of other medical and miscellaneous works, of great and enduring value, a catalogue of which, with prices, will be sent post paid to any one desiring it, and sending full name and post-office address.

VISCOUNTESS AVONMORE has written " A Tale of the Yo-Semite," 296 pp. 12mo, published by Hurd & Houghton. As Mrs. Yelverton has travelled extensively through the great Pacific State, the descriptions given of it, as the story proceeds, and the incidents of the narration, may be considered faithful and lifelike, and doubtless will find many an appreciative reader. The same publishers have issued " Three Successful Girls," being a novel by JULIA CROUCH. $1.50.

"Money, its Christian Use," by the American Tract Society, 50 cents. A book for the times, of great practical value. Also " Heart Life," by T. L. CUYLER, with an admirable portrait of the distinguished author. A book for every thoughtful Christian who wants to keep the heart safe, and obey the Divine injunction, " Give me thy heart." 20 cents. Also, " Summer in the Forest, or, Slender Hands in the Stone Quarries." 304 pp. $1. A beautiful book, and well written ; as also, at same price, "Edna Harrington ; or, the Daughter's Influence in the Home Circle." " The Bugle Call," or, A Summons to Work in Christ's Army, 40 cents. " The Healthy Christian," being an Appeal to the Church, by HOWARD CROSBY, 40 cents.

The United States Patent Laws. " Instructions how to obtain Patents," by MUNN & Co., solicitors of Patents, 37 Park Row, New York, containing also a great amount of valuable information, scientific, practical, and statistical, census for 1870, etc., etc. Price 30 cents.

Temperance. The National Temperance Society, 58 Reade Street, New York, have published "The McAllisters," by Mrs. E. J. RICHMOND ; also " The Seymours," by the author of " The Clinkers," " Purpose," " Paul Vernier," " Jones Clare," etc.

Hydropathy. The same publishers have issued "The Illustrated Hydropathic Encyclopædia," complete in one volume, by R. T. TRALL, M. D. (1,000 pp.) being a system of Hydropathy and Hygiene in eight parts, designed as a guide to families and students, and a text-book for physicians, with numerous engraved illustrations. Dr. Trall is one of the most scientific hydropathists in this country, and this book of his is well worthy of being a standard publication among all those who favor hydropathic treatment, and is a most valuable publication, especially to families in the country who live at a distance from physicians.

"My Wife and I; or, Harry Henderson's History." By HARRIET BEECHER STOWE. 1 vol. 12mo, 474 pp. Illustrated by H. L. Stephens. Price $1.75. Published by J. B. Ford & Co., 39 Park Row, New York. Mrs. Stowe has written nothing so characteristically good for a long time as this delightful novel. It is full of that peculiar charm of her style which completely invests every subject she takes up. She seems gifted with power to create an interest that makes readers eager to get whatever she puts forth in the garb of fiction. "My Wife and I" is her latest, and, in many respects, most thoughtful and complete book. It is eminently a "book for the times," and giving, as it does, Mrs. Stowe's individual ideas about the much vexed *Woman Question*, including marriage, divorce, suffrage, legislation, and all the rights claimed by the clamorous, is spicy enough even for this most *blasé* newspaper age. It has already excited wide-spread interest, so much so that English publishers have arranged for its issue in Great Britain, and it is also in course of translation into French, German, and Swedish.

BEECHER'S "Life of Christ" is published by the same Company, by subscription only. One volume is issued; the second will be published next year; in various styles, from $3 to $15 a volume. It is the great work of Mr. Beecher's life; he has put on it his whole strength; it has been received with much favor by eminent men, and can be read with great pleasure and profit by all Christian people. It is intended to be a faithful portraiture of the life of our Blessed Saviour, in chronological order.

"Parturition without Pain." Wood & Holbrook, 15 Laight Street, New York, have just published for $1.00 a little book of 125 pages, as a Code of Directions for Avoiding the Pains and Penalties of Childbirth. The hints are such as are commended by the highest authorities, and will save women a great deal of suffering. They offer to give it *free* to any person sending $2.00 for their excellent HERALD OF HEALTH.

"The Carriage Painter's Illustrated Manual," containing a Treatise on the Art, Science and Mystery of Coach, Carriage, and Car Painting. Including the Improvements in Fine Gilding, Bronzing, Staining, Varnishing, Polishing, Copying, Lettering, Scrolling, and Oramenting, by F. B. GARDNER. 16mo, cloth. Price $1.00. New York; S. R. Wells, Publisher.

Clear and concise statements of the principal methods in Carriage and Fancy Painting are given. It is thoroughly practical, as the author has had an experience of more than twenty years in the art, and is in every respect a practical painter.

"La Republic." A Political, Scientific, and Literary publication, issued weekly at 42 Broadway, New York, at $5.00 a year, single number, 25 cents; is edited with taste, judgment, and ability, and is commended to the patronage of all the true friends of Cuba, and also to those who are studying that most beautiful of all languages, the pure Castilian.

"London Lancet," monthly, $5.00 a year. Wm. C. Herald, Esq., publisher, 52 John Street, New York. Mr. S. D. Allen is the only Subscription Agent in New York city, Brooklyn, New Jersey, and Pennsylvania.

"Homœopathic Domestic Medicine," by Drs. LAURIE and McCLATCHEY, published by Boericke and Tafel, 145 Grand St., New York, 1034 pages, 8vo, in handsome library style, is the first American, from the twenty-first English edition, to be had from F. E. Boericke, Philadelphia, and 234 Sutter St., San Francisco. It is the most complete and reliable book of Domestic Medicine of its school ever yet published, and is destined to be a standard work and a universal reference book for the rapidly increasing number of families in our land who are falling into that line of practice. It will be sent for five dollars, postpaid, to any one ordering it, accompanying the same with a post-office order, to insure the safe receipt of the money. The same firm is successor to William Radde, and being Homœopathic Pharmaceutists as well as publishers, furnish all the remedies of the school, prepared with nicety and conscientious fidelity. The American editor has with great ability and industry adapted the version to the wants of this country, and incorporated the results of the latest researches and discoveries.

George P. Rowell & Co. are the leading Advertising Agents in the United States, and have the largest advertising establishment in the world; their business integrity is indisputable, and their pecuniary responsibility is A Number 1.

G. P. Putnam & Sons, corner of 4th Avenue and 23d Street, in Association Building, have issued under the title of "Putnam's Handy Book Series," the following useful, practical, and popular books.

1. "What to Eat." A manual for housekeepers. Paper covers, 50 cts.; cloth, 75 cts.
2. "Till the Doctor comes," by GEORGE H. HOPE, M. D. Paper, 30 cts.; cloth, 60 cts. A guide in accidents and sudden illness.
3. "Stimulants and Narcotics," by GEORGE M. BEARD, M. D. Medically, Philosophically, and Morally considered. Paper, 50 cts.; cloth, 75 cts.
4. "Eating and Drinking," by same. In press.
5. "Student's own Speaker," by PAUL REEVES. Paper, 75 cts.; cloth, 90 cts. It is a manual of oratory; patriotic, grave, humorous.
6. "Best Reading." A guide for book-buyers, librarians, household collections, etc. In press.

HALL'S JOURNAL OF HEALTH proper averages twenty-four pages a month, all written by the Editor, who is not responsible for anything outside of these pages, or in the advertisements, which he sees for the first time after they are printed. While the publishers will endeavor to exclude every advertisement unworthy of credit, *Caveat emptor* must be written at the head of each, which means that the purchaser must look out for himself, and if he pays for a rotten apple on the street, or takes a counterfeit copper in the change, there is nobody to blame but himself. The Editor has nothing whatever to do with the business matters of the JOURNAL; he simply writes for it for a compensation, and reads only those letters which ask medical advice. He gives no consultations at his private residence, but may be found at his office, 40 Broadway, Room 44, from ten to one, daily.

